# Circular RNA hsa_circ_0050386 suppresses non-small cell lung cancer progression via regulating the SRSF3/FN1 axis

**DOI:** 10.1186/s12967-023-04812-1

**Published:** 2024-01-12

**Authors:** Jinbin Chen, Boqi Rao, Zeqin Huang, Chen Xie, Yonghui Yu, Binyao Yang, Di Wu, Dedong Wang, Fuman Qiu, Yifeng Zhou, Yibin Deng, Jiachun Lu

**Affiliations:** 1 The State Key Lab of Respiratory Disease, The School of Public Health, Guangzhou Medical University, Guangzhou, 511436 China; 2 Guangzhou Key Laboratory for Clinical Rapid Diagnosis and Early Warning of Infectious Diseases, KingMed School of Laboratory Medicine, Guangzhou Medical University, Guangzhou, 510180 China; 3 Department of Central Laboratory, The 5th Affiliated Hospital of Guangzhou Medical University, Guangzhou, 510700 China; 4 Guangzhou Center for Disease Control and Prevention, Guangzhou, 510440 China; 5 Department of Genetics, Medical College of Soochow University, Suzhou, 215123 China; 6 Centre for Medical Laboratory Science, the Affiliated Hospital of Youjiang Medical University for Nationalities, No. 18 Zhongshaner Rd., Youjiang District, Baise, 533000 China; 7 The State Key Lab of Respiratory Disease, The First Affiliated Hospital, The School of Public Health, Guangzhou Medical University, Xinzao Town, Panyu District, Guangzhou, 511436 China

**Keywords:** Non-small cell lung cancer (NSCLC), Circular RNA (circRNA), Fibronectin 1 (FN1), Serine and arginine-rich splicing factor 3 (SRSF3)

## Abstract

**Background:**

Lung cancer is the most prevalent cancer worldwide, with non-small cell lung cancer (NSCLC) accounting for 85% of all cases. Circular RNAs(circRNA) play crucial roles in regulating the progression of lung cancer. Despite the identification of a large number of circRNAs, their expression patterns, functions, and mechanisms of action in NSCLC development remain unclear.This study aims to investigate the transcriptional expressions, functions, and potential mechanisms of circRNA hsa_circ_0050386 in NSCLC.

**Methods:**

Quantitative real-time polymerase chain reaction (qRT-PCR) was utilized for the analysis of hsa_circ_0050386 expression. Cell proliferation was detected using the IncuCyte Live Cell Analysis System and clone formation assays. Migration and invasion of NSCLC cells were evaluated through Transwell assays. Flow cytometry was performed to assay cell cycle and apoptosis. Western blot was used to investigate protein expression. Protein binding analysis was conducted by employing pull-down assays, RNA immunoprecipitation (RIP), and mass spectrometry. The role of hsa_circ_0050386 in vivo was evaluated through the use of a xenograft model.

**Results:**

The study discovered that hsa_circ_0050386 displayed lower expression levels in NSCLC tissues when compared to adjacent normal tissues. Patients exhibiting lower levels of hsa_circ_0050386 expression exhibited an inverse correlation with the Clinical Stage, T-stage, and M-stage of NSCLC. Functionally, hsa_circ_0050386 suppressed the proliferation and invasion of NSCLC cells both in vitro and in vivo. A comprehensive examination exposed the interaction between hsa_circ_0050386 and RNA binding protein Serine and arginine-rich splicing factor 3 (SRSF3), resulting in the down-regulation of Fibronectin 1 (FN1) expression, which inhibits the progression of NSCLC.

**Conclusions:**

Our study shows that hsa_circ_0050386 suppresses the malignant biological behavior of NSCLC cells by down-regulating the expression of FN1, and may serve as a potential biomarker and therapeutic target for NSCLC treatment.

**Supplementary Information:**

The online version contains supplementary material available at 10.1186/s12967-023-04812-1.

## Background

Lung cancer is the most high-occurring type of cancer and the leading cause of cancer-related deaths worldwide, with non-small cell lung cancer (NSCLC) accounting for 85% of lung cancer cases [1, 2]. Despite the significant development of clinical diagnostic and therapeutic approaches for lung cancer, the 5-year survival rate still ranges from 10 to 20% in most countries [3, 4]. Therefore, it is crucial to investigate the underlying mechanisms of lung cancer to increase our understanding of the disease and to identify possible biomarkers and novel therapeutic targets for early diagnosis and prognosis of lung cancer.

Circular RNAs (circRNA) are a unique type of non-coding RNAs that differ from the conventional linear RNA structure. They have received significant attention in life science research over the past few years [5, 6]. CircRNAs display a covalently closed loop structure and do not adhere to linear RNA's customary 5'–3' polarity, nor do they possess a polyadenylated tail [7]. CircRNAs are characteristically abundant, stable, conserved, and display tissue or developmental-stage specific [8–10]. Their unique properties make circRNAs promising biomarkers or therapeutic targets for various diseases that have been researched. Several studies have emphasized the critical roles of circRNAs in various physiological processes and pathologies such as cardiovascular diseases [11], renal diseases [12], and cancers [13, 14]. CircRNAs functioning as competing endogenous RNA (ceRNA) has been widely reported in various cancers. CircTP63 competes with miR-873-3p and upregulates FOXM1 to promote progression of lung squamous cell carcinoma (LUSC) [15]. Besides, circSATB2 positively regulated FSCN1 expression via binding to miR-326, promoting the proliferation, migration, and invasion of NSCLC [16]. While most studies have put their research interests on the mechanisms of circRNA as miRNA sponges, it is noticeable that circRNAs can also regulate gene expression by interacting with RNA-binding proteins (RBPs) [17]. However, mechanisms of how this interaction between RBPs and circRNAs operates in lung cancer are still unclear.

In this investigation, a particular circRNA, hsa_circ_0050386, encoded by the ANKRD27 gene, was characterized. Analysis of the circRNA expression in NSCLC demonstrated that hsa_circ_0050386 is expressed at lower levels in cancer tissues than in adjacent non-tumorous tissues. Examination of its phenotypic function indicated that hsa_circ_0050386 is capable of inhibiting NSCLC progression from multiple perspectives, thereby positioning it as a possible biomarker and therapeutic target. Interestingly, this observation demonstrates a correlation between the mechanism of hsa_circ_0050386 interaction with the RNA-binding protein, Serine and arginine-rich splicing factor 3 (SRSF3), resulting in the downregulation of Fibronectin 1 (FN1) during NSCLC progression.

## Methods

### Lung cancer tissue samples

A total of 186 samples were collected from hospitals in southern and eastern China. 126 samples were obtained from the Affiliated Hospital of Guangzhou Medical University and 60 were sourced from the Affiliated Hospital of Soochow University. All participants in this study were pathologically confirmed to have primary lung cancer. Comprehensive clinical data, including demographics, clinicopathological metrics, and histopathological information, were recorded (Additional file 2: Table S1). It is noteworthy that none of the subjects had received any kind of anticancer treatment, such as chemotherapy or radiotherapy, before the surgical removal. The study was approved by the Ethics Committee of Guangzhou Medical University(GMU201803010726).

### Cell lines and cell culture

Human lung cancer cell lines H1299, H520, H292, H358, A549, H1795, H460, H1703, and PC9; human bronchial epithelial cell line 16HBE; and human embryonic kidney cell line HEK-293T were acquired from the Cell Bank of Type Culture Collection of the Chinese Academy of Sciences (Shanghai Institute of Cell Biology). H1299, H520, H292, H358, A549, H1795, H460, H1703, and PC9 cells were cultivated in RPMI-1640 medium, including 10% Fetal bovine serum (FBS). 293T cells were cultivated in DMEM medium, including 10% FBS. The cells were maintained in a 37 °C incubator with 5% CO2.

### Microarray analysis

Sample preparation and microarray hybridization were carried out following Arraystar's guidelines. To summarize, we used RNase R (Epicentre, Inc.) to remove linear RNA and enrich circRNAs from total RNA. The enriched circRNA underwent amplification and transcription into fluorescent cRNA with random primer method (Arraystar Super RNA Labeling Kit; Arraystar). The resulting labeled cRNA was then hybridized onto the Arraystar Human circRNA Array V2 (8 × 15 K, Arraystar). After the slide washes, we scanned the arrays using the Agilent Scanner G2505C. Array images were captured using the Agilent Feature Extraction Software (version 11.0.1.1). Quantitative normalization and data assessments were performed using the R software package. We identified circRNAs that were significantly differentially expressed, using a fold change in gene expression criterion that exceeded 2 or was below −2, in addition to an adjusted *P*-value under 0.05.

### RNA and gDNA extraction

Total RNA was extracted from the samples using Trizol (Invitrogen, USA) according to the manufacturer's instructions. Genomic DNA (gDNA) was prepared using the TIANamp Genomic DNA Kit (TIANGEN, China) according to standard procedures.

### Quantitative reverse transcription real-time polymerase chain reaction (qRT-PCR)

The cDNA synthesis was carried out using the PrimeScript RT reagent Kit with gDNA Eraser (Takara, China), followed by qRT-PCR analyses that employed the FastStart Universal SYBR Green Master (Roche, Germany). The primers used in our investigation were sourced from Sangon Biotechnology (Shanghai, China) and are listed in Additional file 3: Table S2.

### RNase R treatment

2 μg of total RNA was incubated for 30 min at 37 °C with or without the addition of 3 U/μg RNase R (GENESEED, Guangzhou). Subsequently, Trizol (Invitrogen, USA) was used to purify the resulting RNA and its quantity was determined by qRT-PCR analysis.

### Fluorescence in situ hybridization (FISH)

The hsa_circ_0050386 probe labeled with Cy3 was synthesized by RiboBio (China). Fluorescent in Situ Hybridization Kit (RiboBio, China) was used to hybridize hsa_circ_0050386 probes to cells. Images were captured using a confocal laser scanning microscope (LSM 880, Zeiss, Germany).

### Nucleocytoplasmic fractionation

RNA was extracted from the nuclear and cytoplasmic fractions using the PARIS kit (Thermo Fisher, USA) according to the manufacturer's instructions. Gene expression was measured by qRT-PCR, with U6 used as the internal reference for the nuclear fraction and GAPDH for the cytoplasmic fraction.

### Plasmid, siRNA, lentiviral construction, and stable transfection

The lentivirus containing hsa_circ_0050386 over-expressing or empty-vector plasmids were produced by GenePharma (Shanghai, China). Plasmids containing small hairpin RNA (shRNA) targeting hsa_circ_0050386 were constructed by GeneCopoeia (Guangzhou, China). According to the instructions, lentivirus-mediated transduction was performed using the Lenti-PacTM HIV Expression Packaging Kit (GeneCopoeia, USA). Stable cell lines were acquired by screening with puromycin and validated through qRT-PCR. Small interfering RNA (siRNA) targeting FN1 and SRSF3 was synthesized by GenePharma (Shanghai, China). GP-transfect-Mate (GenePharma, China) was used for siRNA transient transfection following the instructions provided. The sequences of shRNA and siRNA are listed in Additional file 3: Table S2.

### Cell proliferation assay

1000 cells/well were seeded per well into a 96-well plate, using 100 µl of culture medium supplemented with 10% FBS. The plate was then incubated in an IncuCyte Live Cell Analysis System (Essen Bioscience, UK) at 37 °C with 5% CO_2_. The proliferation of the cells was monitored in real-time using phase-contrast scanning. Cellular confluency in each well was recorded at 2-h intervals for 96 h.

### Colony formation assay

Lung cancer cells that had been treated were placed into 6-well plates at a density of 1000 cells per well and were allowed to incubate for a total of 2 weeks. Following two washes with PBS, the plates were fixed in paraformaldehyde for 30 min. Subsequently, staining was carried out using 0.1% crystal violet for 10 min. The identification of distinct clones, each of which was comprised of more than 50 cells, was achieved via microscopic examination.

### Flow cytometry

Harvested cells were treated by being fixed overnight in ice-cold 75% ethanol at 4 °C, for analysis of the cell cycle. The fixed cells were washed twice with PBS and stained with propidium iodide (PI) (7seapharmtech, China). After staining, cell cycle analysis was performed by FACS scanning flow cytometry. For cell apoptosis analysis, cells at the logarithmic growth stage were harvested and washed twice with PBS. The collected cells underwent staining with Annexin V-FITC and PI (MULTISCIENCES, China) in darkness for 15 min. Following washing, flow cytometry detected apoptosis. The analysis of the data was conducted by FlowJo software (FlowJo).

### Transwell migration and invasion assay

Transwell culture plates (Corning, UK) and BioCoat Matrigel invasion chambers (BD Biosciences) were employed to evaluate cell migration and invasion capabilities. The suspension of cells (1 × 10^5^/mL) was made using a culture medium without FBS. Following this, 100 μL of the suspension was inoculated into the upper Transwell, while 500 μL of medium containing 20% FBS was added to the lower Transwell chamber. After incubation at 37 ℃ for 48 h, the cells in the upper chamber were removed using cotton swabs. The cells in the lower chamber were fixed using a solution of 4% paraformaldehyde and subsequently stained with crystal violet. The mean count of cells that migrated was computed by randomly selecting five fields of view under the microscope (100 ×).

### Xenograft mouse model

BALB/c nude mice, aged 4–6 weeks, were obtained from the Guangdong Medical Laboratory Animal Centre (Guangzhou, China), and later housed under specific pathogen-free (SPF) conditions. The Ethics Committee of Guangzhou Medical University approved this animal experiment.

Each mouse was injected subcutaneously with 5 × 10^6^ cells. Tumor volumes were measured weekly using calipers once discernible. The volume was calculated as follows: volume = length × width^2^ × 0.5. The experimental phase lasted 4 weeks after the initial injection. For the tail vein metastasis model, 1 × 10^6^cells were injected into the mice's tail vein. For the tail vein metastasis model, 1 × 10^6^ cells were injected into the tail vein of nude mice. Treated mice were euthanized when they lost more than 15% of body weight or failed to thrive. Lung tissues were dissected and fixed with 4% Paraformaldehyde Fix Solution and were analyzed by Hematoxylin–eosin (H&E) staining.

### RNA-seq

Total RNA was isolated from A549 and PC9 cells overexpressing hsa_circ_0050386 and corresponding empty vector control cells using the RNeasy Mini Kit (Qiagen, Germany). The sequencing was performed by using the Illumina NovaSeq 6000 platform in SINOTECH GENOMICS (Shanghai, China).

### RNA pull-down assays

The biotinylated probes for hsa_circ_0050386 and the negative control (NC) probes were designed and synthesized by RiboBio (Guangzhou, China). hsa_circ_0050386 probe sequences are listed in Additional file 3: Table S2. The protocol for the RNA pull-down assay was performed based on those previously reported [18].

1 × 10^7^ cells were washed in ice-cold PBS and lysed in 500 μl of co-IP buffer. After that, 3 μg of biotinylated probes targeting the hsa_circ_0050386 back splicing junction region or control probe were added to the cell lysates. The probes in the cell lysates were incubated for 2 h at room temperature. Each binding reaction was then treated with 50 μl of washed Streptavidin C1 magnetic beads (Invitrogen) and left to incubate for one hour at room temperature. The magnetic beads underwent a brief wash cycle of five times using Co-IP buffer. The proteins that were retrieved will be used for mass spectrometry or Western blot analyses.

### RNA immunoprecipitation (RIP)

Following the manufacturer's guidelines, we conducted RIP assays employing the Magna RIP RNA binding protein immunoprecipitation kit (Millipore, USA). For SRSF3 enrichment, we used the Anti-SRSF3 antibody (sc-13510,Santa Cruz Biotechnology) while the Anti-IgG antibody served as a background control. Enriched RNA was quantified using qRT-PCR.

### Western blot analysis

Cells were harvested and lysed on ice with RIPA buffer (Beyotime, China). Next, the lysate was incubated on ice for 15 min and then centrifuged for 15 min (13,000 rpm, 4 °C). The supernatant was collected, and the protein concentration was determined by using the BCA Protein Assay Kit (Beyotime, China). Protein samples (20 μg) were loaded into each lane of the SDS-PAGE lane. Following electrophoresis, proteins were transferred to PVDF membranes blocked with 3% BSA. Protein transferred membranes were incubated with primary antibodies overnight at 4 °C, followed by 1 h of incubation with secondary antibodies at room temperature. Western blots were visualized on a BioSpectrum 515 (Wolf Laboratories Limited, UK) by using the Affinity ECL kit (Affinity, china). The antibodies used in the western blot were: FN1 (1:1000 dilution, Abcam, USA), SRSF3 (1:300 dilution, Santa Cruz Biotechnology, USA), GAPDH (1:5000 dilution, Abcam, USA), HRP-conjugated goat anti-mouse (1:5000 dilution. Beyotime, China), and HRP-conjugated goat anti-rabbit antibody (1:5000 dilution, Beyotime, China).

### Hematoxylin–eosin (H&E) and immunohistochemistry (IHC)

For H&E staining, paraffin-embedded samples were cut into 4 μm thick sections, deparaffinised, rehydrated and then stained with haematoxylin and eosin. In addition, the sections were boiled in citrate buffer (pH = 6.0) for 2 min in an autoclave to recover the antigen. They were blocked in PBS containing 3% BSA for 30 min at room temperature and incubated overnight at 4 °C with antibodies specific for ki67 (1:200, GB111499, Servicebio), PCNA (1:200, GB11010-1, Servicebio) and FN1 (1:100, ab2413, abcam). The DAB method was used for immunoassays.

### Statistical methods

Statistical analysis was conducted using Prism software (GraphPad Software 8). A comparison of the difference between the two experimental groups was performed using Student's t-test, while data of repeated measures were compared using a mixed-effects model. A statistically significant result was defined by a two-sided probability of 0.05 or less. Not significant is represented by 'ns', while *P < 0.05 and **P < 0.01 indicate significance levels.

## Results

### CircRNA hsa_circ_0050386 is downregulated in NSCLC

**Fig. 1:**
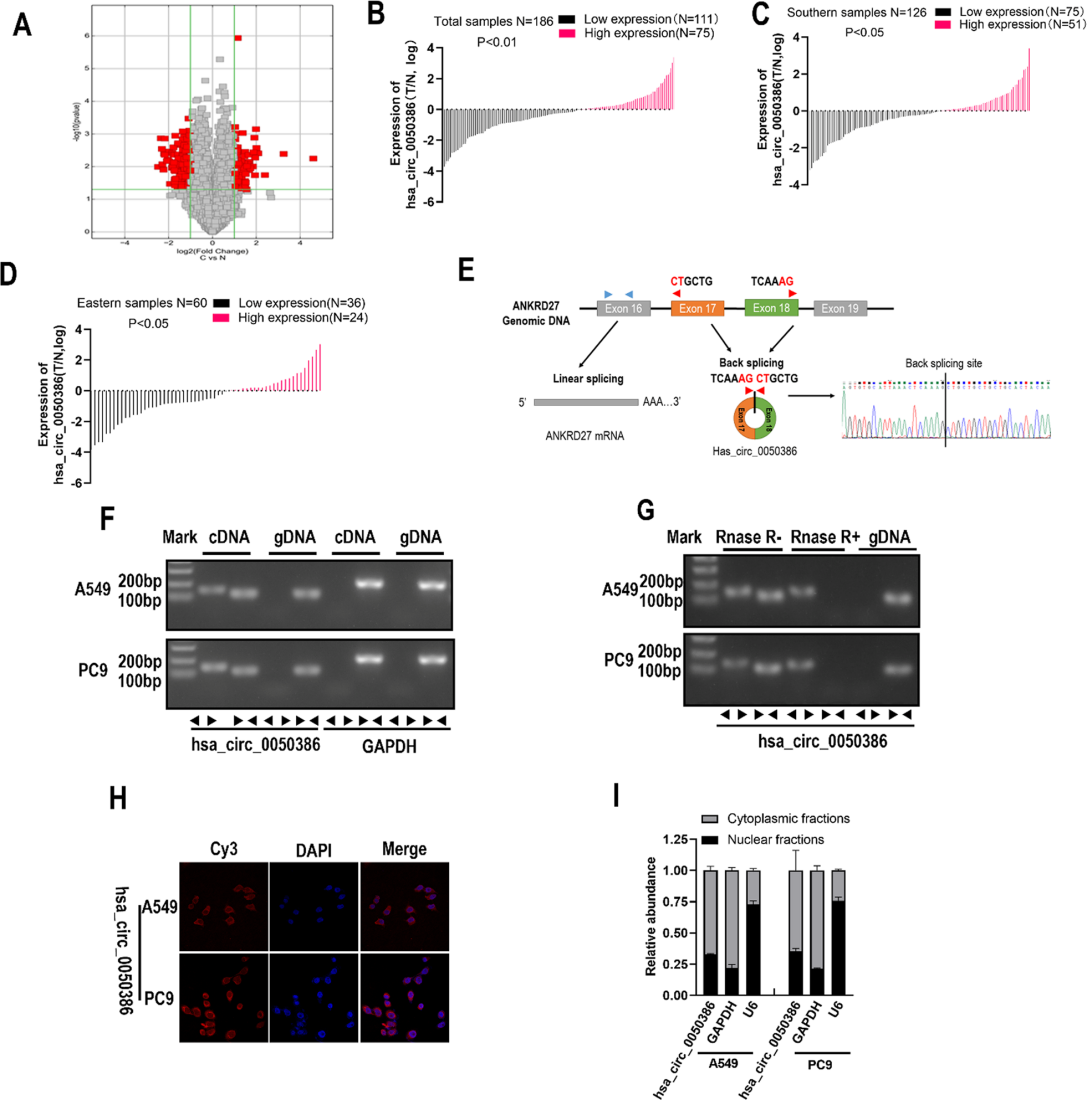
The expression and characterization of hsa_circ_0050386 in lung cancer. **A** volcano plot showing differentially expressed circRNAs in 8 paired lung cancer tissue samples. The cut-off value was defined as fold change > 2 or <  − 2, adjusted *P* < 0.05. **B**–**D** The expression level of hsa_circ_0050386 in lung cancer tissue from two regions in China. High expression was defined as Log2 (T/N expression) value > 0, and low expression was defined as Log2 (T/N expression) value < 0. N, nontumorous tissue; T, tumorous tissue. **E** The schematic showing hsa_circ_0050386 encoded from exon 17,18 of the ANDRD27 gene. **F** PCR and agarose gel electrophoresis detecting hsa_circ_0050386 and GAPDH in cDNA and gDNA by using convergent and divergent primers in A549 and PC9 cells. **G** PCR, and agarose gel electrophoresis detecting hsa_circ_0050386 by convergent and divergent primers in A549 and PC9 cells after treatment with RNase R. gDNA was used as a control. gDNA, genomic DNA. **H** Detection of subcellular location of hsa_circ_0050386 by FISH. Hsa_circ_0050386 probes were labeled with Cy3; Nuclei were stained with DAPI. **I** Detection of the cytoplasmic and nuclear distribution of hsa_circ_0050386 in A549 and PC9 cells by qRT-PCR. GAPDH and U1 were used as positive controls for cytoplasm and nucleus, respectively

The expression of circRNAs was examined in 8 NSCLC tissues and their corresponding non-tumor tissues by using circRNA Microarray. The analysis of differential gene expression identified hsa_circ_0050386 as significantly downregulated (FC = −3.99, *P* < 0.0001) in NSCLC tissues when compared to non-tumor tissues (Fig. 1A). The top 50 up-regulated circRNAs and the top 50 down-regulated circRNAs are listed in the (Additional file 4: Table S3).To further substantiate this finding, the expression of hsa_circ_0050386 was examined in two additional cohorts using qRT-PCR. The study revealed that the expression of hsa_circ_0050386 was significantly lower in 59.5% (75 out of 126) of the NSCLC samples from Southern cohorts and in 60.0% (36 out of 60) of the NSCLC samples from Eastern cohorts. This resulted in an overall downregulation of hsa_circ_0050386 in 59.7% (111 out of 186) of the total samples (Fig. 1B, C, D). Importantly, decreased expression of hsa_circ_0050386 in NSCLC tissues was significantly correlated with larger primary tumor size and advanced clinical stage in NSCLC patients (Table 1). The findings indicate that hsa_circ_0050386 is downregulated in NSCLC and exhibits a negative correlation with advanced clinical stages.

**Table 1:** Correlations between has_circ_0050386 expression and clinical parameters among lung cancer patients

Clinical characteristics	Southern samples N (%)		*P*^*a*^	*P*^*b*^	Eastern samples N (%)		*P*^*a*^	*P*^*b*^	Total N (%)		*P*^*a*^	*P*^*b*^
	Low expression	High expression			Low expression	High expression			Low expression	High expression		
Age												
< 60	45 (57.7)	33 (42.3)	0.593	NA	20 (62.5)	12 (37.5)	0.673	NA	65 (59.1)	45 (40.9)	0.844	NA
≥ 60	30 (62.5)	18 (37.5)			16 (57.1)	12 (42.9)			46 (60.5)	30 (39.5)		
Gender												
Female	51 (58)	37 (42)	0.585	NA	27 (62.8)	16 (37.2)	0.483	NA	78 (59.5)	53 (40.5)	0.954	NA
Male	24 (63.2)	14 (36.8)			9 (52.9)	8 (47.1)			33 (60)	22 (40)		
Tumor family history												
No	69 (61.6)	43 (38.4)	0.178	0.176	31 (57.4)	23 (42.6)	0.219	0.219	100 (60.2)	66 (39.8)	0.652	0.656
Yes	6 (42.9)	8 (57.1)			5 (83.3)	1 (16.7)			11 (55)	9 (45)		
Smoking												
No	27 (54)	23 (46)	0.306	0.133	10 (52.6)	9 (47.4)	0.428	0.425	37 (53.6)	32 (46.4)	0.196	0.166
Yes	48 (63.2)	28 (36.8)			26 (63.4)	15 (36.6)			74 (63.2)	43 (36.8)		
Stages												
I + II	22 (47.8)	24 (52.2)	0.043	0.047	9 (40.9)	13 (59.1)	0.022	0.023	31 (45.6)	37 (54.4)	0.003	0.003
III + IV	53 (66.3)	27 (33.8)			27 (71.1)	11 (28.9)			80 (67.8)	38 (32.2)		
T status												
T1 + T2	36 (49.3)	37 (50.7)	0.006	0.007	13 (50)	13 (50)	0.167	0.168	49 (49.5)	50 (50.5)	0.003	0.003
T3 + T4	39 (73.6)	14 (26.4)			23 (67.6)	11 (32.4)			62 (71.3)	25 (28.7)		
N status												
N0	26 (60.5)	17 (39.5)	0.877	0.866	13 (48.1)	14 (51.9)	0.090	0.083	39 (55.7)	31 (44.3)	0.392	0.391
N1 + N2 + N3	49 (59)	34 (41)			23 (69.7)	10 (30.3)			72 (62.1)	44 (37.9)		
M status												
M0	47 (52.2)	43 (47.8)	0.008	0.010	20 (57.1)	15 (42.9)	0.593	0.555	67 (53.6)	58 (46.4)	0.016	0.016
M1	28 (77.8)	8 (22.2)			16 (64)	9 (36)			44 (72.1)	17 (27.9)		
Histological subtype												
Adenocarcinoma	32 (52.5)	29 (47.5)	0.248	0.202	14 (56)	11 (44)	0.866	0.909	46 (53.5)	40 (46.5)	0.255	0.242
Squamous carcinoma	24 (63.2)	14 (36.8)			12 (63.2)	7 (36.8)			36 (63.2)	21 (36.8)		
Others	19 (70.4)	8 (29.6)			10 (62.5)	6 (37.5)			29 (67.4)	14 (32.6)		

^a^Crude *P* value of Pearson chi square test

^b^*P* value of the binary logistics regression adjusted for variables of age and gender; *NA* not applicable

### Characteristics of hsa_circ_0050386 in NSCLC cells

The circular RNA hsa_circ_0050386 was produced from exons 17 and 18 of the ANKRD27 gene with a total length of 291 nucleotides. To examine its characteristics, we employed divergent primers to amplify the back-splicing junction of hsa_circ_0050386 from DNA products generated using PCR. Subsequently, we sequenced hsa_circ_0050386, with results that indicate a high degree of accuracy and similarity compared to the circRNA identified on the circBase database (http://www.circbase.org/) (Fig. 1E). PCR analysis demonstrated that divergent primers could amplify products from the cDNA library, but not from the genomic DNA (gDNA) library (Fig. 1F). Additionally, the RNase R resistant assay indicated that the hsa_circ_0050386 amplified PCR product from divergent primers was more resistant to RNase R digestion compared to its linear form amplified from PCR using convergent primers (Fig. 1G). To locate hsa_circ_0050386 in A549 and PC9 cells, we conducted nuclear/cytoplasmic fractionation and fluorescence in situ hybridization (FISH). The results indicate that circRNA hsa_circ_0050386 is mainly concentrated in the cytoplasm (Fig. 1H, I).

### Hsa_circ_0050386 inhibits NSCLC progression in vitro

**Fig. 2:**
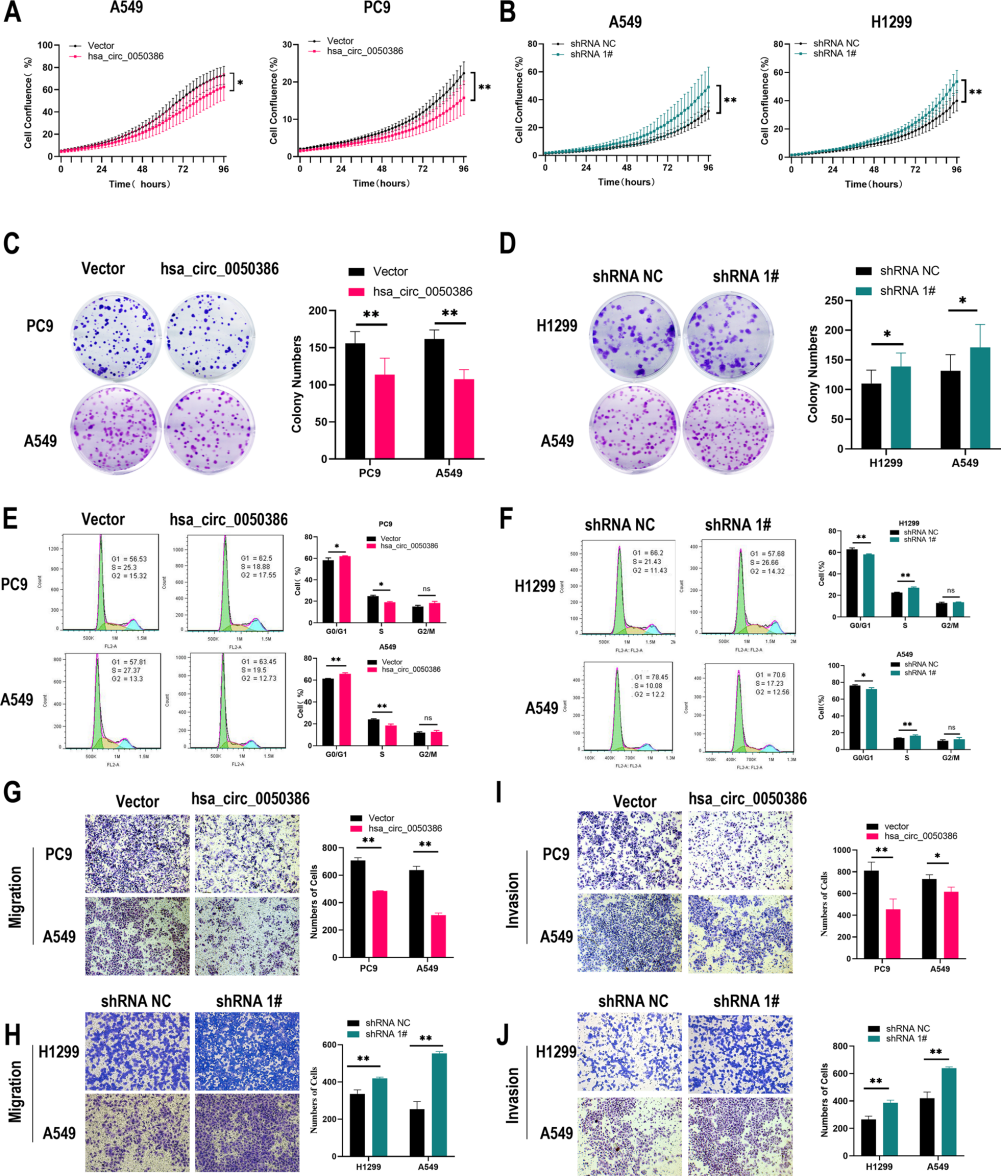
The tumor-suppressive function of hsa_circ_0050386 in vitro. **A**–**B** Cell proliferation kinetics of lung cancer cells with hsa_circ_0050386 overexpression or knockdown detected by incuCyte Live Cell Analysis System. **C**–**D** Colony formation assays detecting the tumor colony forming capacity of lung cancer cells overexpressing or knocking down hsa_circ_0050386. **E**–**F** The effect of hsa_circ_0050386 overexpression or knockdown on NSCLC cell cycle progression assayed by flow cytometry. **G**–**I** Migration and invasion assay for detecting NSCLC cells with hsa_circ_0050386 overexpression or knockdown

To investigate the functions of hsa_circ_0050386 in the progression of NSCLC, we examined the endogenous expression of hsa_circ_0050386 in nine NSCLC cell lines and one normal human bronchial epithelial cell line (16HBE) (Additional file 1: Figure S1A). The expression of hsa_circ_0050386 was found to be downregulated in NSCLC cell lines in comparison to normal human lung cell lines. Based on the expression pattern of hsa_circ_0050386 across cell lines, we conducted loss-of-function assays for hsa_circ_0050386 in H1299 and A549 cells, and gain-of-function assays in PC9 and A549 cells. Additionally, we established a hsa_circ_0050386 stable overexpression cell line in H1299 and A549 (Additional file 1: Figure S1B). For hsa_circ_0050386 knockdown, three shRNAs were designed to knock down hsa_circ_0050386 by targeting the back-splicing junction region. shRNA 1#, shRNA 2# and shRNA 3# could successfully knock down hsa_circ_0050386 expression, and shRNA 1# was highly potent. As we further confirmed that hsa_circ_0050386 did not alter ANKRD27 mRNA expression in NSCLC cells (Additional file 1: Figure S1C), we selected shRNA 1# for hsa_circ_0050386 loss-of-function assays. Proliferation and colony formation assays showed that NSCLC cell growth was significantly suppressed when hsa_circ_0050386 was overexpressed** (**Fig. 2A, C). Consistently, stably silencing the expression of hsa_circ_0050386 promoted cell viability (Fig. 2B, D). Cell cycle assay revealed that stable overexpression of hsa_circ_0050386 increased the number of NSCLC cells in the G0/G1 phase and decreased the number of cells in the S phase, while knockdown of hsa_circ_0050386 resulted in progression of cells from G0/G1 to S phase(Fig. 2E, F). These data suggest that hsa_circ_0050386 could inhibit the cell cycle progression of NSCLC. Furthermore, transwell migration and invasion assays showed that overexpression of hsa_circ_0050386 inhibited NSCLC cell migration, whereas knockdown of hsa_circ_0050386 expression promoted NSCLC cell migration (Fig. 2G–J). Furthermore, hsa_circ_0050386 had no significant effect on cell apoptosis of NSCLC cells (Additional file 1: Figure S1D, E). Taken together, these data suggest that hsa_circ_0050386 may act as a suppressor of NSCLC progression in vitro.

### Hsa_circ_0050386 suppresses NSCLC growth and metastasis in vivo

**Fig. 3:**
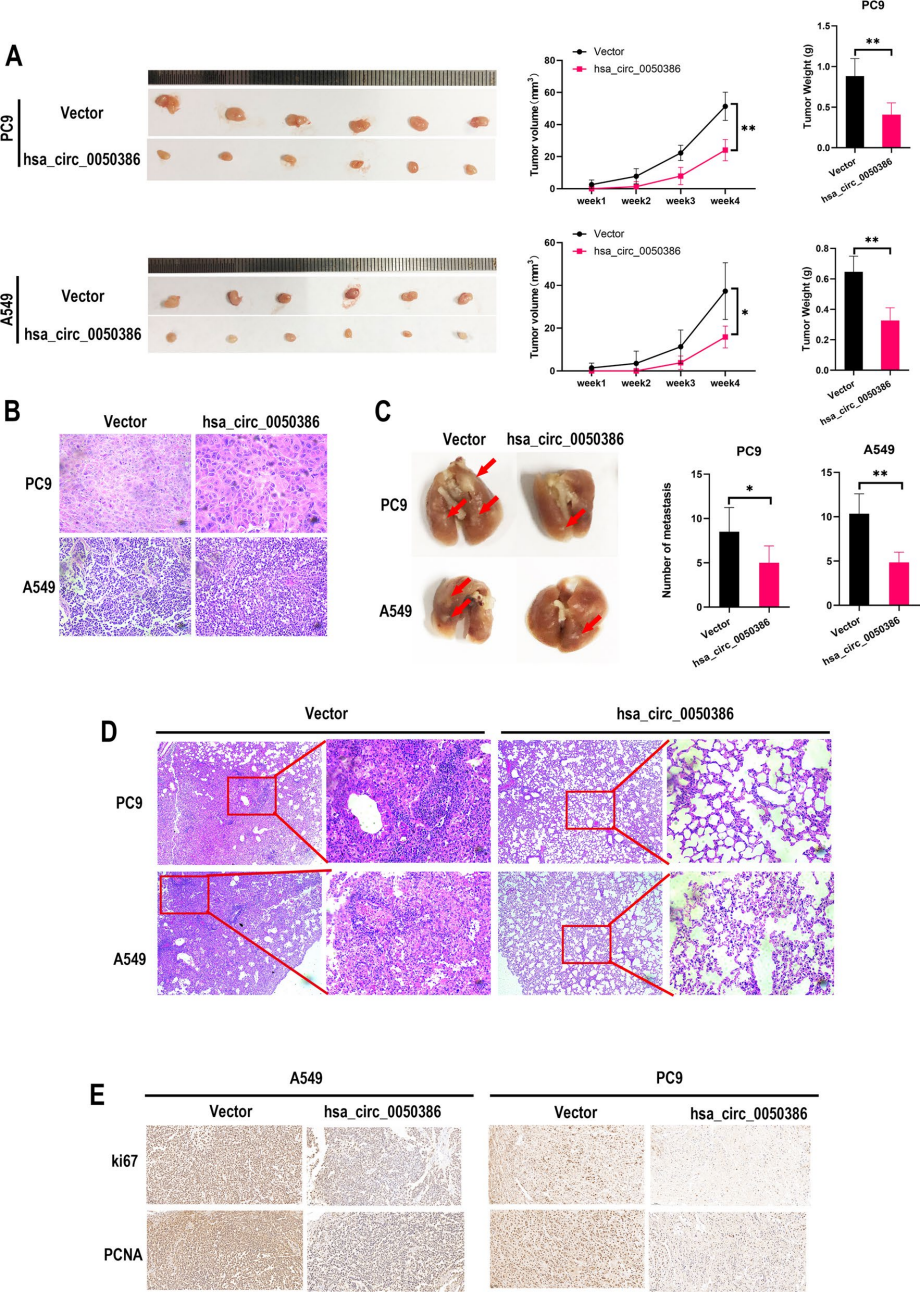
has_circ_0050386 inhibits the progression of lung cancer in vivo. **A** Left panel: The subcutaneous xenograft tumors in nude mice. Middle panel: The volume of the subcutaneous xenograft tumors. Right panel: The weight of the subcutaneous xenograft tumors. **B** H&E staining showing tumor tissue. **C** Representative images and metastasis counts of mouse lung metastasis. **D** H&E staining of mouse lung metastasis. **E** Representative IHC images of Ki67 and PCNA in xenograft tumors tissue. Scale bar, 50 μm

To study the effect of hsa_circ_0050386 on tumor growth and metastasis in vivo, we established a nude mouse xenograft model by inoculating PC9 and A549 cells transduced with either empty vector or hsa_circ_0050386. We observed that overexpression of hsa_circ_0050386 dramatically inhibited tumor growth (Fig. 3A). The composition of the tumor tissue was confirmed by hematoxylin and eosin (H&E) staining (Fig. 3B). Furthermore, tail vein injection of stable NSCLC cells into nude mice indicated that overexpression of hsa_circ_0050386 reduced the number of tumor metastases in the lungs of the mice (Fig. 3C, D). IHC was conducted with ki67 and PCNA on xenograft tissues. The results indicated that the expression levels of ki67 and PCNA were reduced within the tumor tissues that overexpressed hsa_circ_0050386 (Fig. 3E). Taken together, these results demonstrated that hsa_circ_0050386 could inhibit the proliferation, tumourigenicity, and metastasis of NSCLC cells in vivo.

### hsa_circ_0050386 inhibits NSCLC cell progression by regulating FN1

**Fig. 4:**
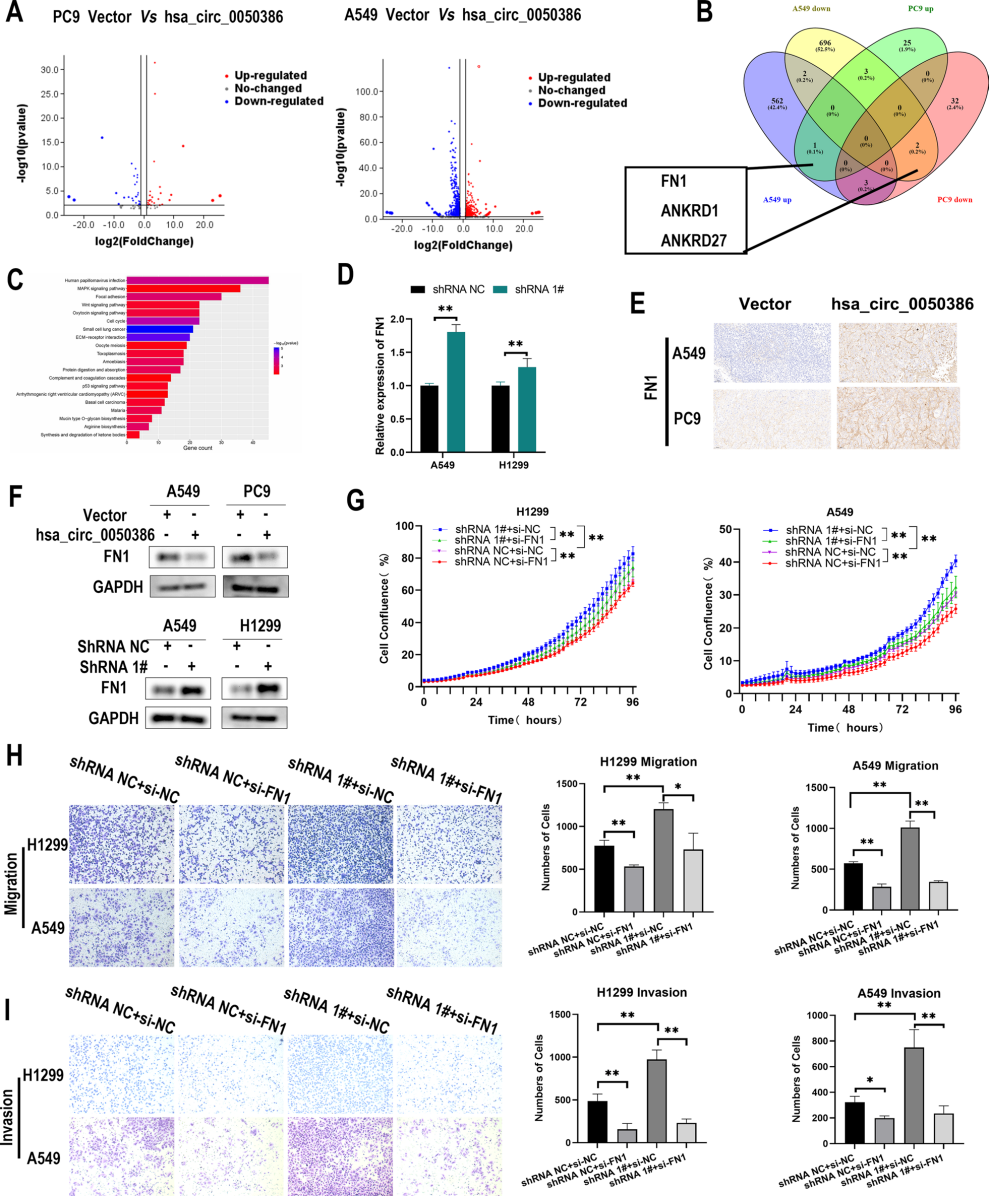
has_circ_0050386 down-regulates FN1 expression and inhibits lung cancer progression. **A** Volcano plot showing the differentially expressed genes upon overexpression of has_circ_0050386 in A549 and PC9 cells. **B** Venn diagram showing the set of mRNAs found to be downregulated or upregulated in both A549 and PC9 cells (filtered by fold change < -2 and adjusted P < 0.05). **C** Pathway enrichment of the differentially expressed genes according to KEGG analysis. **D**, **F** qRT-PCR and Western blot analysis showing relative FN1 expression in lung cancer cells after has_circ_0050386 overexpression or knockdown in cells. **E** Representative IHC images of FN1 in xenograft tumor tissue. Scale bar, 50 μm. **G** IncuCyte Live Cell Analysis System showing the viability of H1299 and PC9 cells stably transfected with shRNA-NC or shRNA 1# or co-transfected with si-NC or si-FN1. **H**–**I** A migration and invasion assay for detecting cell migration and invasion ability of H1299 and PC9 cells stably transfected with shRNA-NC or shRNA 1# or co-transfected with si-NC or si-FN1

To gain further genetic insight into hsa_circ_0050386, we carried out RNA sequencing (RNA-seq) analysis to identify differentially expressed genes (DEGs) in PC9 and A549 cells overexpressing hsa_circ_0050386 in comparison to empty vectors. The findings demonstrated an increase in the expression of 575 genes and a decrease in the expression of 703 genes in A549. Similarly, PC9 showed an increase in expression of 90 genes and a decrease in expression of 37 genes (FC > 2, Padj. < 0.05) (Fig. 4A). Among the differentially expressed genes, ANKRD1 and FN1 were consistently downregulated in PC9 and A549 cells, while ANKRD27 were concordantly upregulated in both (Fig. 4B). KEGG analysis showed that the differentially expressed genes were enriched in "MAPK signaling pathway”, “Focal adhesion", "Wnt signaling pathway", "ECM receptor interaction", etc., which are closely related to lung cancer. It is worth noting that the FN1 of hsa_circ_0050386 belongs to the "Focal adhesion" and "ECM − receptor interaction" pathways(Fig. 4C). To validate this profile observation, the expression of ANKRD1, FN1, and ANKRD27 was detected in H1299 and A549 cells with silenced hsa_circ_0050386 expression using qRT-PCR. The results found that expressions of FN1 were upregulated while the expression of hsa_circ_0050386 was inhibited (Fig. 4D). However, no significant change in the expression of ANKRD1 and ANKRD27 was detected (Additional file 1: Figure S1F, G). Additionally, the western blot analysis supported the reduction of protein expression of FN1 resulting from overexpression of hsa_circ_0050386. This observation is in line with previous findings, The downregulation of FN1 expression was observed upon enhancement of the expression of hsa_circ_0050386(Fig. 4F). We performed IHC with FN1 on xenograft tissues and the staining showed that the expression of FN1 was decreased in the tumor tissues overexpressing hsa_circ_0050386 (Fig. 4E). Therefore, we deemed FN1 as a viable target for regulation by hsa_circ_0050386. Next, we conducted additional research into the role of FN1 in the progression of NSCLC. Our findings indicate that when the most effective siRNAs were utilized to suppress the expression of FN1(Additional file 1: Figure S1H), both the growth of H1299 and A549 cells and the capacity for NSCLC cell metastasis were significantly impeded compared to the mock. Next, perturbation of hsa_circ_0050386 expression did not hinder the significant promotion of proliferation and metastasis in H1299 and A549 cells upon FN1 knockdown (Fig. 4G–I). These results imply that hsa_circ_0050386 might regulate the proliferation and metastasis of NSCLC cells by modulating FN1.

### SRSF3 interacts with has_circ_0050386 and FN1 in NSCLC cells

**Fig. 5:**
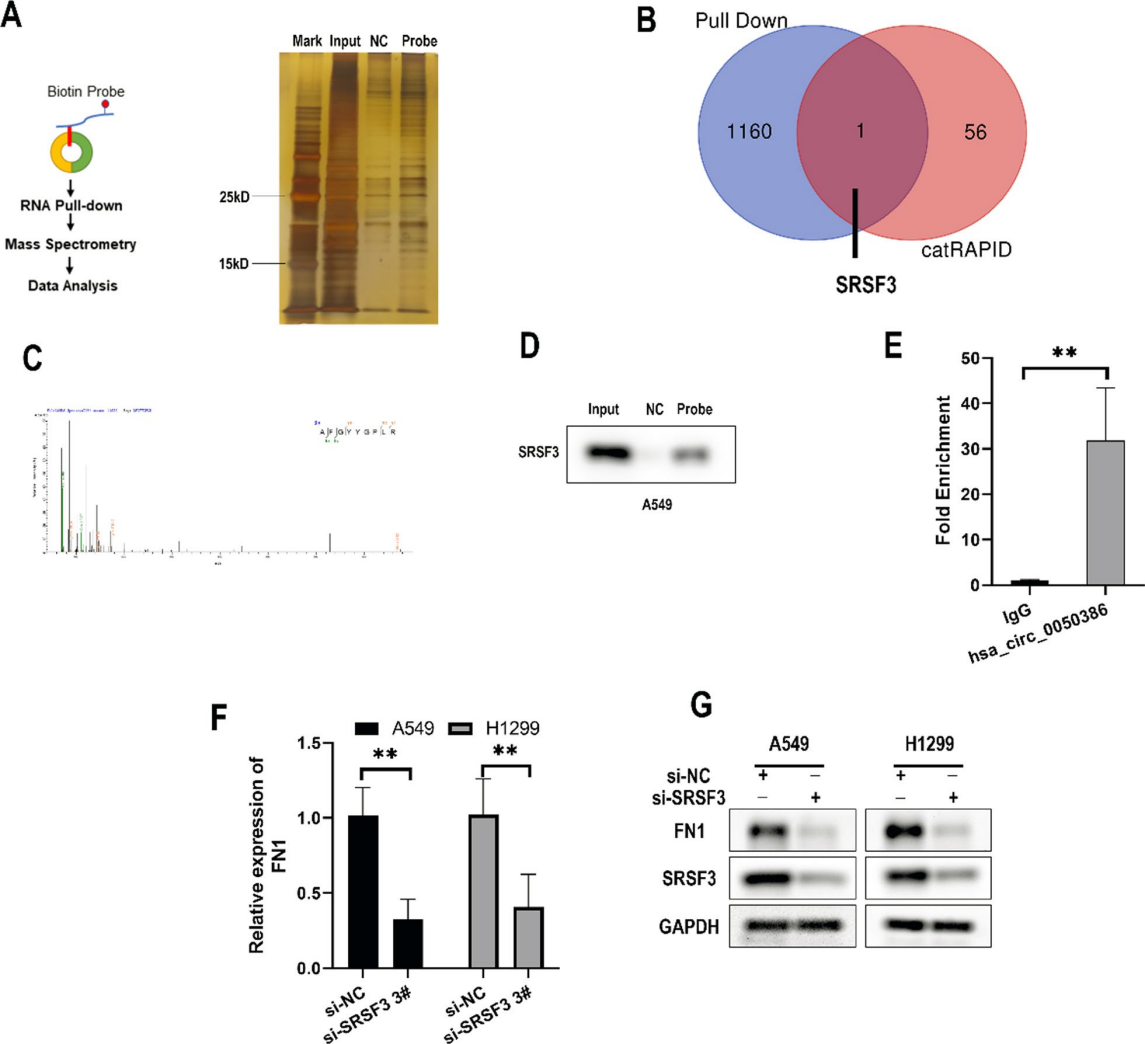
SRSF3 interacts with has_circ_0050386 and FN1 in lung cancer cells. **A** Schematic of biotin-labeled hsa_circ_0050386 and NC probes used for RNA–protein pull-down in A549 cell lysates. A. Silver staining identifying proteins interacting with hsa_circ_0050386. **B** Venn diagram showing proteins identified by mass spectrometry and proteins predicted by catRAPID. **C** Mass spectrometry showing the SRSF3 peptide enriched in the hsa_circ_0050386 probe. **D** Western blot analysis showing protein expression of SRSF3 enriched in hsa_circ_0050386 or NC probe in A549 cell lysates. **E** RNA immunoprecipitation (RIP) assays together with qRT-PCR showed that SRSF3 and hsa_circ_0050386 were enriched using anti-SRSF3 antibody in A549 cell lysates. **F**–**G**. qRT-PCR and Western blot analysis showing the expression of FN1 in A549 and H1299 cells after knocking down the expression of SRSF3 in cells

Recent research suggests that circRNAs may interact with multiple proteins in molecular regulation [19]. To explore the potential mechanism by which hsa_circ_0050386 regulates FN1 expression and whether it functions via protein interaction, RNA pull-down assays were performed along with Liquid Chromatography-Mass Spectrometry (LC–MS). The precipitates in the RNA pull-down assay were separated by SDS–polyacrylamide gel electrophoresis (SDS-PAGE) and subjected to silver staining(Fig. 5A). LC–MS analyses revealed the identification of 1161 proteins associated with has_circ_0050386. Thereafter, we deployed catRAPID tools to predict which proteins may interact with has_circ_0050386. From this collation, protein SRSF3 was projected as a favorable candidate by catRAPID and was also confirmed through LC–MS (Fig. 5B, C). We subsequently conducted a Western Blot and RIP assay to corroborate the observation made (Fig. 5D, E). To investigate whether SRSF3 regulates FN1, we conducted qRT-PCR and western blot analyses to assess FN1 expression in A549 and H1299 cells transfected with SRSF3 siRNA. Our results indicate that the expression of FN1 was lower in si-SRSF3 treated cells than in cells transfected with si-NC (Fig. 5F, G, and Additional file 1: Figure S1I). These findings suggest that SRSF3 could potentially interact with FN1 and has_circ_0050386, which in turn regulates the expression of FN1.

### FN1 is regulated by has_circ_0050386 through SRSF3

**Fig. 6:**
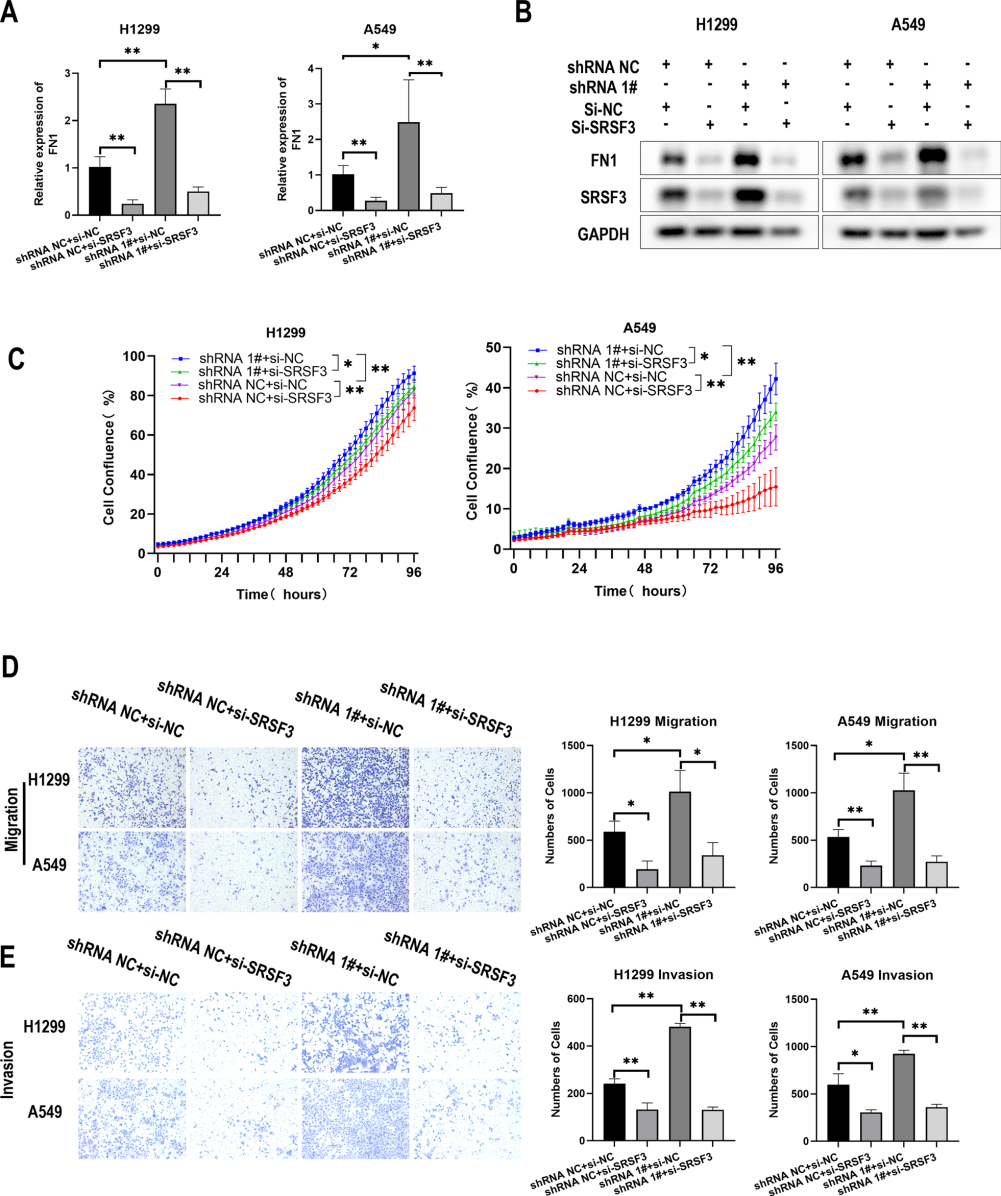
hsa_circ_0050386 inhibits cell proliferation, migration, and invasion of lung cancer via regulating the SRSF3/FN1 axis. **A**–**B** qRT-PCR and Western blot analysis detecting the expression of FN1 in H1299 and PC9 cells stably transfected with shRNA-NC or shRNA 1# or co-transfected with si-NC or si-SRSF3. **C** IncuCyte Live Cell Analysis System detecting viability of H1299 and PC9 cells stably transfected with shRNA-NC or shRNA 1# or co-transfected with si-NC or si-SRSF3. **D**–**E** Migration and invasion assays detecting the cell migration and invasion potential of H1299 and PC9 cells stably transfected with shRNA-NC or shRNA 1# or co-transfected with si-NC

To examine whether has_circ_0050386 regulates FN1 through SRSF3, we assessed the FN1 expression in NSCLC cell lines upon altering the expression of has_circ_0050386 and SRSF3 using qRT-PCR or western blot. The study demonstrates that the silencing of has_circ_0050386 led to a notable elevation in the levels of mRNA and protein expression of FN1 in H1299 and A549 cell lines. However, his effect was not observed in NSCLC cells that were co-transfected with si-SRSF3 RNA. **(**Fig. 6A, B). To confirm the results further, we conducted another rescue experiment. The data indicated that suppressing has_circ_0050386 enhanced the ability of NSCLC cells to proliferate, migrate, and invade. However, no such effect was observed in NSCLC cells that were co-transfected with si-SRSF3 (Fig. 6C, D). These findings imply that hsa_circ_0050386 may inhibit NSCLC cell progression via the SRSF3/FN1 pathway.

## Discussion

CircRNAs represent a new category of non-coding RNAs characterized by a unique closed-loop structure. This striking feature confers greater stability to circRNAs compared to their linear counterparts. Consequently, circRNAs exhibit tremendous promise as both a biomarker and therapeutic target for various afflictions, such as NSCLC [20]. Thousands of circRNAs have been identified in eukaryotes to date. Some of them seem to play a critical role in cancer development and progression through multiple mechanisms of action [21]. The study reveals that has_circ_0050386 is downregulated in NSCLC tissues, and this circRNA can potentially facilitate the suppression of NSCLC cells in vitro and in vivo. Mechanistically, has_circ_0050386 regulates the expression of FN1 and impedes NSCLC progression by interacting with the RNA-binding protein SRSF3.

Clinically, the decreased expression of hsa_circ_0050386 in NSCLC tissues was found to be significantly associated with larger primary tumor size and worse clinical stages in patients. Further experiments proved that hsa_circ_0050386 inhibited cell proliferation and metastasis of NSCLC cells in vitro and in vivo by reducing the expression of FN1. It is noteworthy that FN1 has been determined as a promoter of malignancy in diverse types of cancer, such as breast and colorectal cancer [22, 23]. The interaction of this compound with ITGA5 was found to inhibit apoptosis while promoting viability, invasion, and migration in colorectal cancer. Moreover, it was reported to promote breast cancer progression through interaction with the PI3K/AKT pathway [24]. Consistent with these findings, our research reveals that has_circ_0050386 suppresses the proliferation, migration, and invasion of NSCLC cells through the downregulation of FN1 expression.

The traditional function of circRNAs is to act as "miRNA sponges", leading to their inhibition in miRNA regulation [7, 25]. Nonetheless, the mode of action for most circRNAs remains unclear. The present study provides evidence for the direct mediation of biological functions by hsa_circ_0050386 through binding to the RNA-binding protein SRSF3 and tuning FN1. SRSF3 is a versatile protein that participates in regulating diverse cellular processes, including RNA metabolism, alternative polyadenylation, mRNA export, and transcription termination [26, 27]. Kim et al. discovered that SRSF3 facilitates translational control of p21 mRNA. Depletion of SRSF3 triggers cellular senescence and results in elevated p21 expression, which is independent of p53 [28].

Zhao et al. demonstrated the binding of SRSF3 to mRNA and its involvement in both nuclear and cytoplasmic transportation [29]. As a proto-oncogene, SRSF3 is crucial in cellular proliferation, tumorigenesis, and cancer maintenance [30, 31]. Li et al. discovered that the expression of SRSF3 was higher in pan-cancer tissue in comparison to normal tissue. This overexpression suggested unfavorable outcomes for both overall survival and death-specific survival [32]. Jia et al. discovered that SRSF3 controls alternative splicing of ILF3, thereby stimulating the growth and conversion of cancer cells [33]. The study identified physical interactions between SRSF3 and both FN1 and has_circ_0050386 and a positive correlation between the expression of SRSF3 and FN1. Down-regulating hsa_circ_0050386 can stifle SRSF3 expression and hinder NSCLC cell progression via the SRSF3/FN1 pathway. These findings support previous studies indicating that SRSF3 functions as a proto-oncogene. However, the precise mechanisms by which SRSF3 inhibits the progression of NSCLC in has_circ_0050386 require further investigation in the future.

The topic of the relationship between circRNAs and RNA-binding proteins has been reported in the literature. Studies have revealed that circFOXP1 has an interaction with PTBP1 protein, resulting in the transportation of PTBP1 into the cytoplasm. This interaction leads to the stabilization of PKLR mRNA and in turn, promotes the Warburg effect as well as the progression of tumors [34]. Additionally, the interaction between circNSUN2 and IGF2BP2 increases the stability of HMGA2 mRNA, leading to the promotion of liver metastasis in colorectal cancer [35]. Additionally, circNDUFB2 acts as a protein scaffold to enhance the interaction between TRIM25 and IGF2BPs in non-small cell NSCLC. This leads to the formation of a TRIM25/circNDUFB2/IGF2BPs ternary complex which accelerates the ubiquitination and degradation of IGF2BPs proteins that function as oncogenes [36]. These studies indicate that the interaction with proteins is a crucial mechanism for circRNAs to execute their functions. Our findings are in line with prior research and introduce novel evidence that circRNA interacts with RNA-binding proteins to regulate the expression of target genes.

## Conclusions

In conclusion, we have discovered a new circRNA, has_circ_0050386, that shows decreased expression levels in NSCLC. This circRNA has been found to impede NSCLC advancement by interacting with SRSR3 and lowering FN1 expression.

### Supplementary Information

**Additional file 1. Figure S1.** Supplementary figure.

**Additional file 2: Table S1.** Clinical characteristics among lung cancer patients.

**Additional file 3: Table S2-1.** List of primers for qPCR. **Table S2-2.** List of probe sequences for RNA pulldown. **Table S2-3.** List of sequences for siRNAs. **Table S2-4**. List of Target sequences for shRNA.

**Additional file 4. Table S3.** List of top 50 differentially expressed circRNAs

## Data Availability

Data sets used during the current study were available from the corresponding authors upon reasonable request.

## Abbreviations

NSCLC: Non-small cell lung cancer
circRNA: Circular RNA
FN1: Fibronectin 1
SRSF3: Serine and arginine-rich splicing factor 3
FISH: Fluorescence in situ hybridization
FBS: Fatal bovine serum
siRNA: Small interfering RNA
shRNA: Small hairpin RNA
