# Supplementary figures and images for: Circular RNA hsa_circ_0050386 suppresses non-small cell lung cancer progression via regulating the SRSF3/FN1 axis
Source: J Transl Med. 2024 Jan 12;22:47. doi: 10.1186/s12967-023-04812-1 (PMC10785521; doi:10.1186/s12967-023-04812-1)

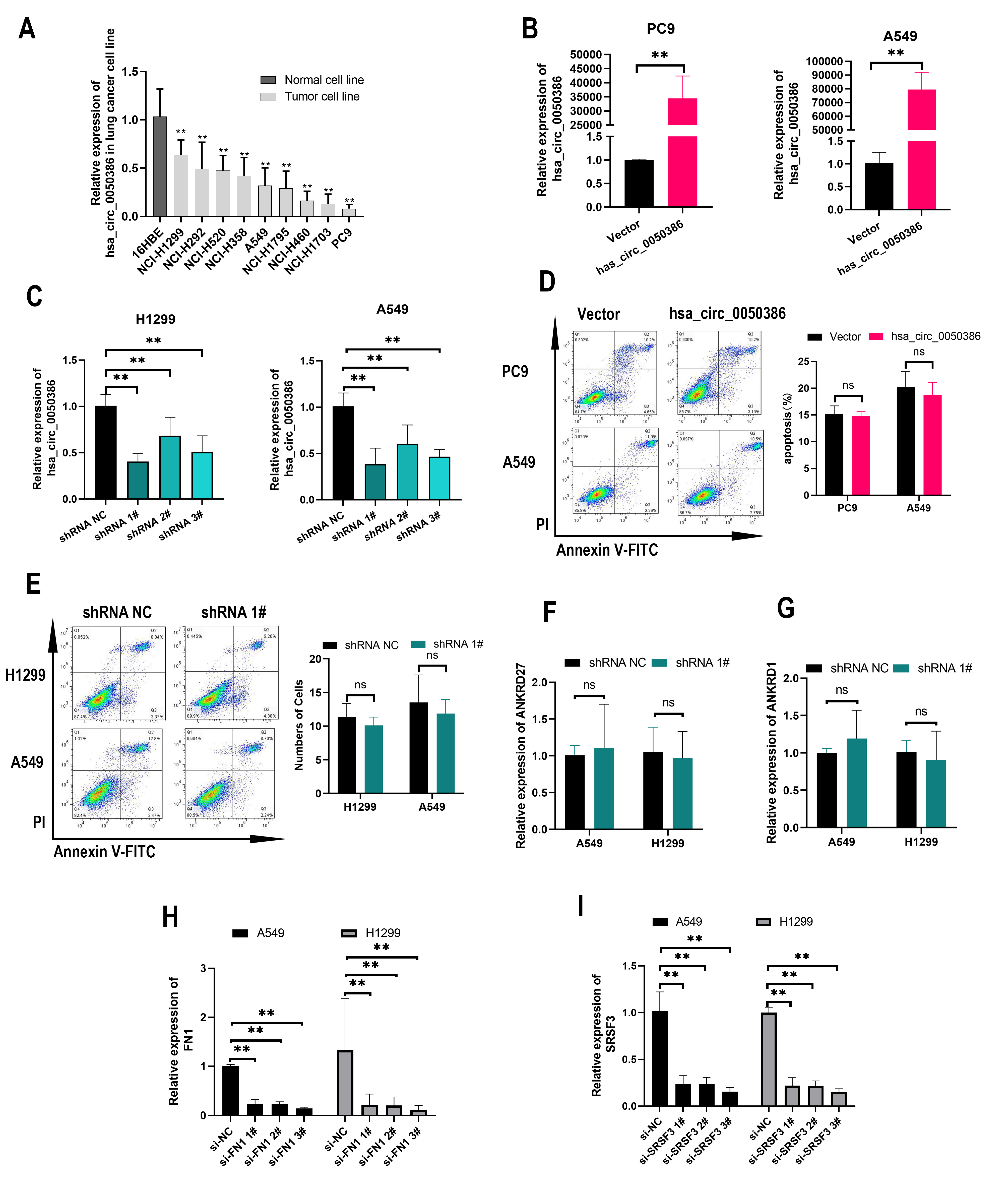

Supplement: Supplementary file 1 — Additional file 1. Figure S1. Supplementary figure. [file 12967_2023_4812_MOESM1_ESM.tif]
