# Supplementary material for: Circular RNA hsa_circ_0050386 suppresses non-small cell lung cancer progression via regulating the SRSF3/FN1 axis
Source: J Transl Med. 2024 Jan 12;22:47. doi: 10.1186/s12967-023-04812-1 (PMC10785521; doi:10.1186/s12967-023-04812-1)
Supplement: Supplementary file 2 — Additional file 2: Table S1. Clinical characteristics among lung cancer patients. [file 12967_2023_4812_MOESM2_ESM.docx]

| **Additional Table. S1 Clinical characteristics among lung cancer patients** | | | | | | |
| --- | --- | --- | --- | --- | --- | --- |
| **Clinical characteristics** | **Southern samples N(%)** | **Eastern samples N(%)** | | **χ^2^** | ***P^a^*** | **TotalN(%)** |
| **Total** | 126(67.7) | 60(32.3) |  | |  | 186(100) |
| **Age** |  |  |  | |  |  |
| <60 | 78(61.9) | 32(53.3) | 1.236 | | 0.266 | 110(59.1) |
| ≥60 | 48(38.1) | 28(46.7) |  | |  | 76(40.9) |
| **Gender** |  |  |  | |  |  |
| Female | 38(30.2) | 17(28.3) | 0.065 | | 0.799 | 55(29.6) |
| Male | 88(69.8) | 43(71.7) |  | |  | 131(70.4) |
| **Tumor Family History** |  |  |  | |  |  |
| No | 112(88.9) | 54(90) | 0.052 | | 0.819 | 166(89.2) |
| Yes | 14(11.1) | 6(10) |  | |  | 20(10.8) |
| **Smoking** |  |  |  | |  |  |
| No | 50(39.7) | 19(31.7) | 1.119 | | 0.290 | 69(37.1) |
| Yes | 76(60.3) | 41(68.3) |  | |  | 117(62.9) |
| **Stages** |  |  |  | |  |  |
| I+II | 46(36.5) | 22(36.7) | 0.000 | | 0.983 | 68(36.6) |
| III+IV | 80(63.5) | 38(63.3) |  | |  | 118(63.4) |
| **T status** |  |  |  | |  |  |
| T1+T2 | 73(57.9) | 26(43.3) | 3.482 | | 0.062 | 99(53.2) |
| T3+T4 | 53(42.1) | 34(56.7) |  | |  | 87(46.8) |
| **N status** |  |  |  | |  |  |
| N0 | 43(34.1) | 27(45) | 2.047 | | 0.152 | 70(37.6) |
| N1+N2+N3 | 83(65.9) | 33(55) |  | |  | 116(62.4) |
| **M status** |  |  |  | |  |  |
| M0 | 90(71.4) | 35(58.3) | 3.162 | | 0.075 | 125(67.2) |
| M1 | 36(28.6) | 25(41.7) |  | |  | 61(32.8) |
| **Histological Subtype** |  |  |  | |  |  |
| Adenocarcinoma | 61(48.4) | 25(41.7) | 0.913 | | 0.634 | 86(46.2) |
| Squamous Carcinoma | 38(30.2) | 19(31.7) |  | |  | 57(30.6) |
| Others ^b^ | 27(21.4) | 16(26.7) |  | |  | 43(23.1) |
| *^a^* Chi-square test； | | | | | | |
| *^b^* Large cell lung cancer, small cell lung cancer and other mixed or undifferentiated lung cancers. | | | | | | |
