# Supplementary material for: Circular RNA hsa_circ_0050386 suppresses non-small cell lung cancer progression via regulating the SRSF3/FN1 axis
Source: J Transl Med. 2024 Jan 12;22:47. doi: 10.1186/s12967-023-04812-1 (PMC10785521; doi:10.1186/s12967-023-04812-1)
Supplement: Supplementary file 3 — Additional file 3: Table S2-1. List of primers for qPCR. Table S2-2. List of probe sequences for RNA pulldown. Table S2-3. List of sequences for siRNAs. Table S2-4. List of Target sequences for shRNA. [file 12967_2023_4812_MOESM3_ESM.docx]

| **Additional Table. S2-1.List of primers for qPCR** | | |
| --- | --- | --- |
| Gene | Sequence(5’-3’) | |
| hsa_circ_0050386 | Forward | AGTGTGCATTAAACTCAAAGCTG |
|  | Reverse | AACCAGAGCCTTCACACAGT |
| GAPDH | Forward | GGTATCGTGGAAGGACTCATGAC |
|  | Reverse | ATGCCAGTGAGCTTCCCGTTCAG |
| FN1 | Forward | CGGTGGCTGTCAGTCAAAG |
|  | Reverse | AAACCTCGGCTTCCTCCATAA |
| ANKRD1 | Forward | AGTAGAGGAACTGGTCACTGG |
|  | Reverse | TGTTTCTCGCTTTTCCACTGTT |
| ANKRD27 | Forward | CTCATCGACCTCCTGGTTTCC |
|  | Reverse | GTCACGCTCTGGTAGCCCTT |
| SRSF3 | Forward | TGGCAACAAGACGGAATTGGA |
|  | Reverse | CAAAGCCGGGTGGGTTTCTA |

| **Additional Table.S2-2. List of probe sequences for RNA pulldown** | |
| --- | --- |
| Gene | Sequence(5’-3’) |
| hsa_circ_0050386 (5’biotin) | /5bio/AGCAGCTTTGAGTTTAATGC |
| NC(5’biotin) | /5bio/ATAGAAGGATTTGCTCGTCT |

| **Additional Table.S2-3 List of sequences for siRNAs** | | |
| --- | --- | --- |
| Gene | Sequence(5’-3’) | |
| Si-FN1 1# | sense | GCAGCACAACUUCGAAUUATT |
|  | antisense | UAAUUCGAAGUUGUGCUGCTT |
| Si-FN1 2# | sense | GGAGAUGAGUGGGAACGAATT |
|  | antisense | UUCGUUCCCACUCAUCUCCTT |
| Si-FN1 3# | sense | GCAAGCAGCAAGCCAAUUUTT |
|  | antisense | AAAUUGGCUUGCUGCUUGCTT |
| Si-SRSF3 1# | sense | CAUCGUGAUUCCUGUCCAUTT |
|  | antisense | AUGGACAGGAAUCACGAUGTT |
| Si-SRSF3 2# | sense | GCUAGAUGGAAGAACACUATT |
|  | antisense | UAGUGUUCUUCCAUCUAGCTT |
| Si-SRSF3 3# | sense | CCCUCGAGAUGAUUAUCGUTT |
|  | antisense | ACGAUAAUCAUCUCGAGGGTT |

| **Additional Table.S2-4 List of Target sequences for shRNA** | |
| --- | --- |
| Gene | Target Sequence(5’-3’) |
| Sh-circ_0050386 1# | AAACTCAAAGCTGCTGCTG |
| Sh-circ_0050386 2# | TAAACTCAAAGCTGCTGCT |
| Sh-circ_0050386 3# | GCATTAAACTCAAAGCTGC |
